# Blistering Collodion

**Published:** 1849-11

**Authors:** 


*Blistering Collodion.*—A mixture of collodion with cantharides has been contrived as a substitute for the ordinary blistering plaster. The cantharides are digested in the ether, and the latter afterwards mixed with the gun-cotton. The part to be blistered is painted over with the collodion by a pencil.—*London Lancet.*

